# Supplementary material for: Effects of indium exposure on respiratory symptoms: a retrospective cohort study in Japanese workers using health checkup data
Source: PeerJ. 2020 Jan 15;8:e8413. doi: 10.7717/peerj.8413 (PMC6969551; doi:10.7717/peerj.8413)
Supplement: Supplemental Information 3 [file peerj-08-8413-s003.docx]

**Appendix file**

The subjects and items of indium medical examination according to the Ordinance on Prevention of Hazards due to Specified Chemical Substances.

**The subjects of indium medical examination**

A worker who is constantly engaged in the production and/or handling of the indium.

**The items included indium medical examination**

1. Survey of work history
2. Simple survey of working conditions
3. Past history of subjective symptoms such as cough, nasal drip, and shortness of breath caused by indium compounds
4. Presence or absence of subjective symptoms such as cough, throat, and shortness of breath
5. Serum indium
6. Serum KL-6
7. Chest X-ray imaging or examination by special X-ray imaging (limited at the time of hiring or reassignment)

Note:

1. “Simple survey of working conditions” includes changes in working conditions since the last special health check, information on the concentration of the substance in the environment, working time, frequency of exposure, distance from the substance, and use of respiratory protective equipment. This survey is conducted mainly by a doctor listening to the worker. In addition, there is a method to ask the health supervisor of the workplace in advance about the indium concentration in the environment.
2. “Presence or absence of subjective symptoms such as cough, throat, and shortness of breath” include smoking history.
3. “Examination via special X-ray imaging” refers to examination via CT (computed tomography).
